# P80 Identifying extended surgical prophylaxis in a cohort of elective post-operative critical care patients

**DOI:** 10.1093/jacamr/dlag102.086

**Published:** 2026-06-26

**Authors:** J Cavlin, S Gillen, P McTavish, M MacLeod, C McCue

**Affiliations:** Intensive Care Unit, Glasgow Royal Infirmary, Glasgow, Scotland; Pharmacy Department, Glasgow Royal Infirmary, Glasgow, Scotland; Intensive Care Unit, Glasgow Royal Infirmary, Glasgow, Scotland; Pharmacy Department, Glasgow Royal Infirmary, Glasgow, Scotland; Microbiology Department, Glasgow Royal Infirmary, Glasgow, Scotland; Intensive Care Unit, Glasgow Royal Infirmary, Glasgow, Scotland

## Abstract

**Background:**

Antimicrobial resistance (AMR) remains a critical global health challenge, with intensive care units (ICUs) representing a high-risk environment for the emergence and propagation of resistant organisms. Factors such as high antibiotic utilization, patient acuity, invasive interventions, and prolonged hospitalization contribute to this risk. Effective antimicrobial stewardship, including optimization of surgical antibiotic prophylaxis, is therefore essential in ICU settings to minimize unnecessary antibiotic exposure while maintaining patient safety (Luyt et al., 2014). National guidance from the Scottish Antimicrobial Prescribing Group (SAPG) emphasizes that surgical antibiotic prophylaxis should generally not extend beyond a single post-operative dose, and if continued, the indication must be clearly documented.

**Objectives:**

To characterize the patient cohort receiving extended post-operative surgical prophylaxis and identify compliance with guidelines and areas for quality improvement.

**Methods:**

This study evaluated the prevalence and patterns of extended surgical prophylaxis (ESP) among elective post-operative patients admitted to Glasgow Royal Infirmary ICU between January and June 2025. Patients were identified using our national audit system (Wardwatcher), and data collected on demographics, outcomes, post-operative documentation and antibiotic utilization.

**Results:**

A total of 105 patients were identified, with 102 included in the final analysis after exclusion of emergency cases. Among these, 62 patients (61%) received antibiotics for any indication, and 40 patients (39%) received ESP. Documentation of an antibiotic plan was present in 68 cases (67%), meeting the SAPG standard in just over two-thirds of patients, though gaps remained. Of the 40 patients receiving ESP, 36 were documented in post-operative instructions, while 4 had no documentation. Only 11 of these cases aligned with existing local guidance (splenectomy/sarcoma with flap reconstruction), while the majority—25 cases (69%)—received ESP outside of Greater Glasgow and Clyde (GGC) guidance. Furthermore, where guidance existed, 73% (8/11) of patients received antibiotics for a duration exceeding the recommendation. ESP prescribing without guideline support was most prevalent in urology (83% of cases within the specialty), followed by general surgery and orthopaedics. Antibiotic prescribing patterns revealed a total of 118 antibiotic episodes (prophylaxis and treatment) across the cohort. Notably, 34 patients had no documentation of antibiotic indication, including 8 prophylaxis and 4 ESP episodes, demonstrating ongoing challenges in adherence to stewardship principles. Outcome analysis showed that patients receiving any antibiotics had longer median ICU (7 versus 4 days) and hospital lengths of stay (16 versus 8 days) compared to those not receiving antibiotics. Patients receiving ESP had intermediate outcomes, with a median ICU stay of 5 days and hospital stay of 14.5 days. The proportion of positive microbiology cultures with resistant organisms was higher in patients receiving antibiotics overall (32%) compared to those without antibiotics (3%), though lower in the ESP subgroup (17.5%). This particular result is confounded by availability of specimens for analysis being weighted to the antibiotic received cohort.

**Conclusions:**

In conclusion, ESP is commonly prescribed in our cohort, unfortunately often outwith existing local guidance. These findings highlight significant opportunities to improve compliance with SAPG standards, reduce unnecessary antibiotic exposure, and strengthen antimicrobial stewardship practices in the ICU setting.

